# Risk factors predicting prognosis and outcome of elderly patients with isolated traumatic brain injury

**DOI:** 10.1186/s13018-018-0975-y

**Published:** 2018-11-03

**Authors:** Roman C. Ostermann, Julian Joestl, Thomas M. Tiefenboeck, Nikolaus Lang, Patrick Platzer, Marcus Hofbauer

**Affiliations:** 1 Department of Orthopaedics and Trauma Surgery, Division of Trauma Surgery, Medical University of Vienna, Waehringer Guertel 18 – 20, A – 1090 Vienna, Austria; 2 Department of Orthopeadics and Trauma Surgery, Division of Trauma Surgery, St. Vincent Hospital, Shoulder & Sports Clinic, Medical University of Vienna, Baumgasse 20A, 1030 Vienna, Austria; 3 Department of Trauma Surgery, University Hospital of St. Poelten, Karl Landsteiner University of Health, St Pölten, Austria

**Keywords:** Elderly patients, Isolated severe traumatic brain injuries (TBI), Respiratory failure, Outcome prediction

## Abstract

**Background:**

Traumatic brain injury (TBI), particularly in the elderly patient population, is known to be the single largest cause of death and disability worldwide. The purpose of this retrospective study was to evaluate clinical factors predicting poor outcome with special emphasis on the impact of respiratory failure (RF) on mortality in elderly patients with isolated severe TBI.

**Methods:**

All elderly patients (age ≥ 65 years) with isolated severe head injury, admitted to this level I trauma center, during a period of 18 years (from January 1992 to December 2010) were identified from the trauma registry. The medical records were reviewed for demographics, mechanism of injury (MOI), GCS score at admission, RF, pupillary light reflex (LR), CT findings (subdural hematoma, subarachnoid hematoma, edema, midline-shift), and whether there was conservative treatment or surgical intervention and the Glasgow Outcome Score (GOS) at hospital discharge. Stepwise logistic regression analysis was used to identify risk factors for a poor prognosis and outcome.

**Results:**

The following variables influenced the mortality: respiratory failure, pupillary response, and the injury severity score (ISS). A significant increased risk of death was also found for patients with a midline shift of over 15 mm.

**Conclusions:**

The present study predicts a strong correlation between respiratory failure, pathological pupillary response, a higher ISS, and substantial midline shift with poor outcomes in elderly patients sustaining an isolated severe TBI.

**Trial registration:**

Clinical trials: ID: NCT02386865. Registered 12 March 2015—retrospectively registered.

## Introduction

Traumatic brain injury (TBI), particularly in the elderly patient population (age ≥ 65 years of age), is known to be the single largest cause of death and disability worldwide [1, 2]. Considering the rise in life expectancy and the availability of advanced and more effective treatments for chronic diseases, TBI, resulting in approximately 66,000 deaths annually in Europe [3], is a growing serious public health concern.

While TBI is less common in the elderly patient population, their impact and severity contributes significantly to long-term morbidity and mortality. The reason for the increased mortality in this age population compared to younger adults was pointed out in previous studies. Reasons like co-morbidities, reduced physical reserves, and histories of polypharmacia as factors influencing the outcome were reported [4–8]. The length of hospital stay and subsequent need for rehabilitation following severe TBI are significantly higher in elderly patients, thus highlighting the increased morbidity and costs for managing this patient group [2]. Therefore, early patient characteristics at the emergency department (ED) such as hemodynamic parameters and pupillary response, which potentially predict good or poor outcome following TBI, are becoming invaluable for clinical decision-making, family counseling, evaluation of quality of treatment, and medical resource allocation. Although several valuable studies and prognostic models for outcome prediction [2, 9] are primarily focusing on the Glasgow Coma Scale (GCS), the Abbreviated Injury Scale (AIS) and computed tomography (CT) results exist, and to date, no study is available describing the potential impact of respiratory failure (RF) and secondary neurological decline during ED management on mortality and outcome in the elderly patient population with isolated severe TBI.

The purpose of this study was to evaluate prognostic factors for poor outcome and especially to investigate the role of RF that occurred during ED treatment on mortality in elderly patients with isolated severe TBI. Our hypothesis was that patients, who sustained RF and neurological decline during ED treatment for isolated severe TBI, would show a higher mortality and poor outcome compared to patients, who did not present with these symptoms. We consider this specific information to be essential to physicians requiring early decision making aids at the ED to determine which elderly patient with isolated severe TBI would most likely benefit from aggressive surgical intervention or immediate care unit (ICU) treatment.

## Material and methods

The study was approved by our institutional review board and has been registered in a publicly accessible registry (clinicaltrials.gov ID: NCT02386865).

### Patient population

All adult patients with isolated severe TBI, admitted to this level I trauma center, during a period of 18 years (from January 1992 to December 2010) were identified from the prospectively gathered trauma registry. The inclusion criteria for this study were (1) patients aged 65 years and older and (2) acute severe TBI defined by an Abbreviated Injury Scale (AIS-head) score of ≥ 3 for the head region. Exclusion criteria for this study were as follows: (1) oral intubation prior to ED admission, (2) no details of the time of injury (e.g., chronic subdural hematomas), and (3) any concomitant injuries. Patients who received oral intubation prior to ED admission were excluded due to the high variability of indications other than RF or secondary neurological decline (e.g., oral intubation required for aspiration protection) that may not be necessarily related to TBI-induced secondary RF. The medical records of all patients who met the inclusion criteria were reviewed for age, sex, mechanism of injury (MOI), GCS score at admission, RF, pupillary light reflex (LR), CT-findings (subdural hematoma, subarachnoid hematoma, edema, midline-shift), and whether there was conservative treatment or surgical intervention and the Glasgow Outcome Score (GOS) at hospital discharge.

### ED management

At ED admission, all patients were evaluated by an in-house trauma team, staffed by two trauma surgeons, two anesthesiologists, and two emergency nurses. All patients received the same standardized treatment protocol, according to the Advanced Trauma Life Support guidelines [10]. Acute RF was defined as a life-threatening condition, characterized by (1) objective signs of irregular breathing patterns (extreme dyspnea, shortness of breath, or difficult breathing) and (2) a PaO2 value of less than 60 mmHg while breathing air, or a PaCO2 of more than 50 mmHg that required mechanical ventilation. Medical management was provided according to the Guidelines for Management of Severe Head Injury. The control of the cerebral perfusion pressure included oxygenation (fraction of inspired O_2_ 40–100%), head elevation, fluid resuscitation, sedatives (short-acting benzodiazepines, e.g., midazolam (1–2 mg/h), or narcotics, e.g., fentanyl (25–100 μg/h, mild hyperventilation (PCO_2_ 30–33 mm HG), and osmotherapy (0.25 g/kg mannitol every 4–6 h) or hypertonic saline (3% boluses of 250 to 500 ml) up to a serum osmolality of 320 mOsm/kg H_2_0. All patients received a CT scan evaluation, and the scan was repeated 6 and 24 h after injury, as well as immediately after surgical intervention.

### Clinical outcome

Clinical outcome at the time of hospital discharge was assessed using the GOS as described by Jennet et al. [11]. The GOS measures global functioning as a combination of neurological functioning and dependence on others with five outcome categories: (1) death, (2) persistent vegetative state, (3) severe disability (conscious but dependent on others for daily activities), (4) moderate disability (disabled but independent in daily activities), and (5) good recovery (normal life resumed, with minor neurological deficits possible).

### Statistical analysis

Descriptive statistics were calculated in order to get an overview of the data. To analyze the impact of several risk factors on the occurrence of the event (dead: NO vs. YES), the first univariate analyses (chi-square tests, Fisher exact tests, *t* tests, Wilcoxon tests) were performed. A stepwise logistic regression was done for the occurrence of the event accounting for all univariate significant parameters. Calculated statistical significance (*p* value) is reported. According to the univariate tests, the risk factors respiratory failure, subdural hematoma, subarachnoid hematoma, edema, pupils, midline shift as well as age, GCS, AIS-head, and ISS were selected for the stepwise logistic regression. All analyses were done using the SAS 9.1 System.

## Results

During the 16-year period, 596 elderly patients were admitted with the diagnosis of isolated severe TBI to this level I trauma center. Of this cohort, 96 patients received oral intubation prior to ED-admission, 67 patients had oral intubation following ED-arrival for elective surgical interventions of associated injuries, and 106 patients sustained isolated concomitant injuries, which were treated non-operatively. Twenty-eight patients arrived with multiple injuries, and seven patients were lost to follow-up. These patients were excluded, leaving 292 patients who met the inclusion criteria.

There were 126 (43.2%) males and 166 (56.8%) females with a mean age of 80.8 years (range, 65.3 to 99.4 years). Patient’s demographic and clinical characteristics are shown in Table 1. Alcohol consumption and anticoagulants showed no significant influence on mortality, with (*p* = 0.686) and (*p* = 0.059), respectively. The tested variables, RF, patients’ age, pupillary LR, AIS, ISS, and the GCS score at admission were all significantly associated with mortality (*p* < 0.05). Detailed information concerning initial CT findings is depicted in Table 2. Due to the obvious lack of correlation between outcome and sex, injury mechanism, transportation method, alcohol, and anticoagulants, these factors were excluded as candidate variables for further analysis by stepwise logistic regression.

**Table 1:** Patient demographic and clinical characteristics at admission

Variables	Survived *N* = 215 (%)	Death *N* = 77 (%)	*p* value
Male	89 (70.6)	37 (29.4)	
Female	126 (75.9)	40 (24.1)	0.3116
Age^1^	79.8 (8.7)	83.4 (7.7)	0.0015
GCS score at the ED^2^	14.1 (3–15)	10.9 (3–15)	< 0.0001
AIS-head^1^	3.39 (0.58)	4.39 (0.71)	< 0.0001
ISS^1^	11.8 (4.8)	19.8 (5.9)	< 0.0001

*GCS* Glasgow Coma Scale, *ISS* Injury Severity Score, *AIS* Abbreviated Injury Scale

^1^Mean (SD)

^2^Median (range)

**Table 2:** Incidence of CT abnormalities on initial head CT

CT characteristics	Survived *n* = 215 (%)	Death *n* = 77 (%)	*p* value
SDH			0.0230
Yes	46 (32.4)	96 (67.6)	
No	31 (20.7)	119 (79.3)	
EDH			0.9005
Yes	9 (27.3)	24 (72.7)	
No	68 (26.3)	191 (73.6)	
SAH			0.007
Yes	38 (35.5)	69 (64.5)	
No	39 (21.1)	146 (78.9)	
Hemorrhagic contusion			0.1550
Yes	47 (29.7)	111 (70.3)	
No	30 (22.4)	104 (77.6)	
Edema			< 0.0001
Yes	26 (57.8)	19 (42.2)	
No	30 (22.4)	104 (77.6)	
Midline shift			< 0.0001
No	22 (10.1)	197 (89.9)	
< 0.5 cm	5 (50)	5 (50)	
0.5–1.5 cm	31 (72.1)	12 (27.9)	
> 1.5 cm	19 (95)	1 (5)	
Basal cisterns			< 0.0001
Open	36 (14.4)	214 (85.6)	
Partial open	20 (100)	0	
Closed	21 (100)	0	

*SDH* subdural hematoma, *EDH* epidural hematoma, *SAH* subarachnoid hematoma

According to the univariate tests, the risk factors RF, subdural hematoma, subarachnoid hematoma, edema, pupillary LR, midline shift as well as age, GCS score, AIS, and ISS were selected for stepwise logistic regression. The variable “basal cisterns” was not included in the logistic regression due to a 100% mortality rate for patients being affected by this factor. Finally, the logistic regression analysis found a significant influence of respiratory failure (*p* = 0.0005) on the mortality rate (Table 3). An odds ratio of 9.396 (95%, CI 2.657–33.220) indicates that patients sustaining RF during early ED management had a significantly higher risk of death. Detailed information of patients with RF is presented in Table 4. Furthermore, a significantly higher risk of mortality was found in patients with pathological pupillary LR and a higher ISS as shown in Table 3. A significantly greater risk of death was also found in patients presenting with midline shifts greater than 15 mm as compared to patients who showed no midline shift (*p* < 0.0001). The additional risk factors, initially significant in the univariate analysis did not prove to be significant in the multiple models. The incidence of major CT pathologies is shown in Table 2. A subarachnoid hemorrhage or contusion was found in 265 (90%) patients, and midline shifts in 73 (25%) patients. The midline shift, edema, subdural hematoma and basal cistern injuries were significantly associated with poor outcome (*p* < 0.05).

**Table 3:** Results of multivariate logistic regression analysis

Variables	*p* value	OR	95% CI
Respiratory failure	0.0005	9.396	2.657–33.220
Pupillary reaction	0.0212	3.393	1.200–9.594
ISS	< 0.0001	1.179	1.098–1.267

*OR* odds ratio, *95% CI* 95% confidence interval, *ISS* Injury Severity Score

**Table 4:** Characteristics of patients with respiratory failure

Variables	Respiratory stable *n* = 270 (%)	Respiratory unstable *n* = 29 (%)	*p* value
Mortality	54 (20.5)	23 (79.3)	< 0.0001
Age^1^	81 (8.7)	78 (7.9)	n.s
GCS score at the ED^2^	13.6 (3–15)	10.2 (3–15)	< 0.0001
AIS-head^1^	3.6 (0.7)	4.3 (0.8)	< 0.0001
ISS^1^	13.3 (5.7)	19.2 (6.3)	< 0.0001

*GCS* Glasgow Coma Scale, *ISS* Injury Severity Score, *AIS* Abbreviated Injury Scale, *n.s* not significant

^1^Mean (SD)

^2^Median (range)

Two hundred forty-one patients were treated non-operatively with a mortality rate of 12.4% (30 patients) while 42 patients merely underwent palliative treatment due to their hopeless situation. A total of nine patients underwent neurosurgical treatment. Seven craniotomies and two hole trepanations were performed with a mortality rate of 71% (five patients) and 50% (one patient), respectively. The functional outcome, measured by the GOS at the time of hospital discharge revealed 77 (26.4%) patients who died, 18 patients (6.2%) in a persistent vegetative state, 32 (11%) with severe disability, 24 (8.2%) with a moderate disability, and 141 (48.3%) with a good recovery at discharge.

## Discussion

Due to associated co-morbidity factors, pre-existing polypharmacia and reduced physical reserves, elderly patients (age over 65 years) sustaining isolate severe TBI have been reported to expect increased mortality rates and poor neurological recovery compared to their younger counterparts [2, 5, 6, 8]. With the increase of life expectancy, improved health services and more active lifestyles, TBI, especially in the elderly population, is a significant public health problem of growing importance. Beside the goal to survive the initial trauma, the “gold” measure of outcome following severe TBI should primarily consider the functional status of post-rehabilitation patients, as survival, often in a permanent vegetative state or unable to be independent in daily life, is an enormous emotional and financial burden to the rehabilitation staff as well as families. The decision, to which patient may benefit from early aggressive and maximal surgical treatment e.g. Decompressive Craniotomy (DC) remains controversial in the current literature [12, 13]. Although several studies and models for outcome prediction exist, no studies are available describing the impact of RF during ED management on mortality and outcome in elderly patients with severe TBI. The purpose of this cohort study was to evaluate predictive factors for poor outcome and to investigate the role of acute RF during treatment on mortality of elderly patients with isolated severe TBI.

It is recognized that advanced age is one of the main prognostic factors and has a significant influence on mortality after TBI [2, 8]. A meta-analysis of four prospective series of patients with severe TBI reported that only 15% of elderly patients (age > 65 years) had a favorable outcome at 6 months post-rehabilitation [14]. Kilaru et al. [4] showed in their retrospective study of elderly patients with TBI an overall survival rate of 27%, after an average of 38 months post-rehabilitation. Pompucci et al. [15], in a series of 55 patients, showed advanced age as an independent predictive factor for worse outcome (*p* = 0.005) in patients older than 65 years. According the Indications Guidelines of Guerra et al. [12], DC should only be performed in patients under the age of 50 years. However, the reason for poor functional outcome remains multifactorial. The higher incidence of chronic concomitant diseases (e.g., stroke, cancer, coronary artery disease) and their respective needs for medication such as anticoagulants are very likely contributing factors [16]. Furthermore, the aging brain seems to be more sensitive to ischemia, [17] and offers an impaired regenerative capacity [18]. Additionally, several studies have shown a low-admission GCS, especially in elderly patients, associated with significantly higher mortality rates and poor outcome [2, 4]. Cagetti et al. [10] reported a 100% mortality rate in elderly patients (aged > 80 years), with a GCS score of 11 or less. Mitra et al. [19] showed a poor outcome, e.g., death or persistent vegetative state at a GCS score of 3 to 5 in 100%, at a score of 6 to 7 in 67%, and at a score of 8 in 20%. After the first univariate analyses, our results also revealed the GCS as a strong predictive factor of mortality. Patients who survived had a significantly (*p* < 0.0001) higher GCS score at admission compared to those who died. However, treatment discussions regarding aggressive management or palliative care cannot be solely based on the admission’s GCS score. The GCS is known to be affected by numerous factors unrelated to TBI, such as hypotension, hypoxia, alcohol or drug intoxication, and medical sedation or paralysis [2]. In contrast, the AIS-head classification can provide some guidelines to be used for decision-making. A higher AIS-head score is associated with an increased mortality.

Although several prognostic models have been developed to predict outcome for patients with severe TBI, actually, the impact of RF on mortality in these patients is scarce in the current literature. Acute RF, in the context of TBI, is a life-threatening condition resulting from the secondary impact directly to the head injury. The bleeding and consecutive swelling of the brain can cause an increased intracranial pressure, thus, in the absence of acute medical care, ultimately leads to an incarceration of the respiratory center of the brainstem. Therefore, we find the RF constituting the final syndrome of distinct brain swelling and beginning of incarceration to be an exceptionally important and convincing parameter for the prediction of outcome in elderly patients with TBI. Our logistic regression analysis found a significant (*p* < 0.0005) influence of RF on the mortality rate. Of 29 elderly patients with isolated TBI and RF, 23 (79.3%) died during ED management. This patient population also showed a significant higher ISS and lower GCS compared to respiratory stable patients.

Previous reports regarding the evaluation and scoring of CT scanning, determining the severity of TBI, found the presence of midline shift to be associated with unfavorable outcomes [20–22]. Jacobs et al. [23] found the limit to be 15 mm before death and poor outcome followed. Nelson et al. [24], in their extended analysis of 861 TBI patients, found the midline shift as the most important parameter for prediction of favorable or unfavorable outcome. Consistent with those findings, our study showed a significant (*p* < 0.0001) higher risk of death for patients with a midline shift > 15 mm compared to patients without a midline shift.

Although survival following trauma is the immediate goal, more emphasis should be placed on the quality of life for the survivors. Kilaru et al. [4], in their retrospective study of severe TBI, *n* = 40 elderly patients (age > 65 years) found a survival rate of 27% at a mean follow-up of 38 months. Again, the admission GCS score and neurological deficits were strong predicting factors for mortality. In the low GCS (3 to 8) range, the probability of death or vegetative state was 100% at a GCS score of 3 to 5, 67% at a score of 6 to 7, and 20% at a score of 8. In a meta-analysis of four prospective studies of 5600 patients with severe traumatic brain injury, Hukkelhoven et al. [14] reported only 15% of elderly patients (age > 64 years) had a favorable outcome after 6 months. In our group of elderly patients, which incurred tube intubation due to RF secondary to neurological worsening, only 3 (7.3%) patients were discharged with an assessment of good recovery while 204 (88.8%) patients without RF declined to achieve the same.

The main strength of this study is the relatively large number of included patients and the fact that all patients were treated by practically the same trauma staff at the level I trauma center of central Europe’s biggest hospital. The retrospective design of the study and the fact that no long-term home stay evaluation of the clinical outcome was performed, however, represent the most apparent limitations.

## Conclusion

The current study shows an association between RF, pathological pupillary LR, a higher ISS and degree of midline shift with poor outcomes in elderly patients, and isolated severe TBI. Particularly, the reduction of RF appears to be a solid indicator for mortality and should be considered when assessing elderly patients with isolated TBI. However, the necessity for further research regarding prognostic factors following TBI in elderly patients is essential as the average age of our population, the current environment of cost reduction, and the numbers of elderly patients affected by TBI all continue to advance.

## Abbreviations

AIS: Abbreviated Injury Scale
CT: Computed tomography
ED: emergency department
GCS: Glasgow Coma Scale
GOS: Glasgow Outcome Scale
ICU: Immediate care unit
ISS: Injury severity score
LR: Light reflex
MOI: Mechanism of injury
RF: Respiratory failure
TBI: Traumatic brain injury
